# *In Vitro* Study on Antihypertensive and Antihypercholesterolemic Effects of a Curcumin Nanoemulsion

**DOI:** 10.3797/scipharm.ISP.2015.05

**Published:** 2016-02-14

**Authors:** Heni Rachmawati, Irene Surya Soraya, Neng Fisheri Kurniati, Annisa Rahma

**Affiliations:** 1 School of Pharmacy, Bandung Institute of Technology, Ganesah 10, Bandung 40132, Indonesia; 2 National Research Center for Nanoscience and Nanotechnology, Bandung Institute of Technology, Ganesah 10, Bandung 40132, Indonesia

**Keywords:** ACE Inhibitor, Curcumin, Self nanoemulsion, HMG-CoA reductase inhibitor, UV

## Abstract

Atherosclerosis and hypertension can potentially progess into dangerous cardiovascular diseases such as myocardial infarction and stroke. Statins are widely used to lower cholesterol levels while antihypertensive agents such as captopril are widely prescribed to treat high blood pressure. Curcumin, a phenolic compound isolated from *Curcuma domestica*, has been proven effective for a broad spectrum of diseases, including hypertension and hypercholesterolemia. Therefore, curcumin is quite promising as an alternative therapeutic compound. Our previous studies have proven a significant increase in physical properties, bioavailability, and stability of curcumin when encapsulated in a nanoemulsion. The purpose of this study was to assess the ability of the nanoemulsion in enhancing curcumin activity as a antihypertensive and antihypercholesterolemic agent. The formulation and preparation method of the curcumin nanoemulsion have been developed in our previous study. Physical characterization was performed, including measurement of droplet size, polidispersity index, zeta potential, entrapment efficiency, and loading capacity. Antihypertensive activity of curcumin was evaluated by determining Angiotensin Converting Enzyme (ACE) inhibition *in vitro*. A substrate for ACE, *hippuryl-L-histidyl-L-leucine* was allowed to react with ACE, resulting in hippuric acid formation as the product. The degree of ACE inhibition by curcumin was represented by the amount of hippuric acid formed. Antihypercholesterolemic activity of curcumin was studied using the HMG-CoA reductase assay equipped with a 96-well UV plate. This assay was based on the spectrophotometric measurement of the decrease in absorbance which represents the oxidation of NADPH by the catalytic subunit of *3-hydroxy-3-methylglutaryl-CoA reductase* (HMGR) in the presence of the substrate HMG-CoA. Curcumin is known to have no significant difference in inhibiting ACE compared to Captopril, but when it was incorporated in the self-nanoemulsifying carrier, it slightly increased the inhibitory effect on ACE. In contrast, the effect of curcumin in reducing cholesterol based on the HMGR assay was more pronounced. Curcumin encapsulated in a nanoemulsion showed significant cholesterol-lowering activity compared to a standard drug, pravastatin. Therefore, we conclude that curcumin does not show ACE inhibitory effects, but has potential use as an alternative therapeutic compound to treat hyperlipidaemia. Curcumin encapsulated in a nanoemulsion increased not only the HMGR inhibition, but also ACE inhibition of curcumin. These effects are suggested to be the result of improved solubility in the nanoemulsion system.

## Introduction

Socio-economic, environmental, and demographic changes, when people have adopted unhealthy lifestyles, such as smoking, lack of physical activity, high fat and calories intake, and alcohol consumption are thought to be a risk factor for non-communicable diseases, particularly cardiovascular disorders. Hypertension and hypercholesterolemia are the major factor of morbidity and mortality problem that occurs today.

Hypertension is a condition of elevated blood pressure greater than or equal to 140/90 mmHg. Angiotensin Converting Enzyme (ACE) inhibitors is a drug class that is commonly used in treating hypertension. Some of the most popular ACE drugs are enalapril, benazepril, ramipril, and captopril. ACE inhibitors work by preventing the changes of angiotensin I to angiotensin II and inhibiting the conversion of bradykinin into inactive metabolites.

Atherosclerosis is a disease caused by the accumulation of fat in the walls of arteries. This event often develops into heart disease and stroke [1]. Statins, the metabolite derived from *Aspergillus terreus* currently become the main drug prescribed to lower cholesterol levels [2]. HMG-CoA reductase inhibitors lower cholesterol level by increasing low density lipoprotein (LDL) receptor in the hepatocyte membrane thereby increasing the elimination of LDL.

However, the use of synthetic drugs constantly gives unfavorable effects to the body. This has driven researchers to conduct numerous studies in order to find a safer alternative medicine. One of the study is regarding the potential activity of compounds derived from medicinal plants.

Curcumin is a compound derived from turmeric or *Curcuma domestica*. Curcumin has proven its effect in several pathological conditions including cancer, inflammation, obesity, and cardiovascular diseases [3]. In hypertensive heart failure, curcumin can reduce systolic dysfunction and inhibit the hypertrophy [4]. Curcumin also has been reported to lower cholesterol and improve heart plasma lipoprotein levels. Some studies also show a decrease in the risk of hypercholesterolemia by curcumin in animals induced by atherogenic diet [2].

However among its many benefits, curcumin has unfavorable properties, which are high lipophilicity and poor bioavailability after oral administration [3]. Therefore many efforts were made to improve the value of curcumin, including formulation of curcumin into a spontaneous nanoemulsion system. Previous research has shown a significant increase in physical properties, bioavailability, and stability of curcumin in nanoemulsion carrier system.

The aim of this study was to examine the potential effects of nanoemulsion system in improving antihypertensive and antihypercholesterolemic activities of curcumin *in vitro*. The examination of antihypertensive and antihypercholesterolemic was carried out using ACE assay kit and HMGR assay kit, respectively. The activities were compared to standard drugs – captopril and pravastatin, and pure curcumin in aqueous solution. It was expected that curcumin in nanoemulsion form would show the prospects as an alternative therapeutic agent in hypertension and hyperlipidemia therapy in the future.

## Results and Discussion

### Spontaneous Nanoemulsification

Spontaneous formation of nanoemulsion can be achieved by carefully selecting the oil phase, surfactant, co-surfactant, and their ratio. In this study, curcumin nanoemulsion was prepared using formulation and method developed by Rasaputri (2010) [5]. Glyceryl monooleate (GMO) was chosen as the oil phase due to its medium carbon chain, which is more favorable in spontaneous nanoemulsification compared to other oils with longer carbon chain. Curcumin has a high oil/water partition coefficient (log P = 3.1) and should be dissolved in the oil phase. GMO as oil, Tween 20 as surfactant, and polyethylene glycol 400 (PEG 400) as cosurfactant with a ratio of 1: 8: 1 provided sufficient protection for oil droplets. Tween 20 has hydrophilic-lipophilic balance (HLB) value of 16.7 [6] in which recommended in the formulation of Self Nanoemulsion (SNE). In addition, Tween 20 has a branched alkyl structure that can penetrate the structure of the oil to form a layer of surfactant and thus, SNE films can be formed [7]. The use of PEG 400 as cosurfactant together with a surfactant was intended to reduce interfacial tension in order to obtain a stable globule formation [8].

In this study, nanoemulsion preparation used mechanical vibration by applying ultrasonic waves on the mixture. This process induces micrometer-sized particle to break into nanometer-sized particle. As the ultrasonication process progressed, the temperature of the mixture increased due to high energy produced. Evaporation or degradation of the sample might occur. Therefore, the temperature was maintained by cooling the sample on ice bath during ultrasonication.

### Droplet Size and Size Distribution

**Fig. 1:**
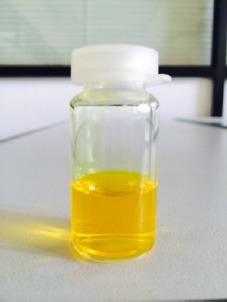
The visual appearance of curcumin nanoemulsion

The average diameter of droplets in curcumin nanoemulsion was 42.93 ± 29.85 nm. Visually, the nanoemulsion was clear, yellowish, and transparent, as shown in Figure 1. The transparent appearance indicated that the droplet size was much smaller than the optical wavelength of the visible spectrum, causing a very weak light scattering.

In the previous studies, the amount of curcumin that can be incorporated into the nanoemulsion system was limited. The more curcumin added to the oil phase, the larger the droplet size [9]. When the oil phase is not able to dissolve the active substance, the particles will be excluded from the globule and this affects the determination of droplet size.

Curcumin nanoemulsion has a low polydispersity index value of 0.36 ± 0.04. Polydispersity index represents the uniformity of the particle size/globules in the preparation. The lower the polydispersity index value, the higher uniformity of globule size in the dosage form [10].

### Morphology of nanoemulsion globule

**Fig. 2:**
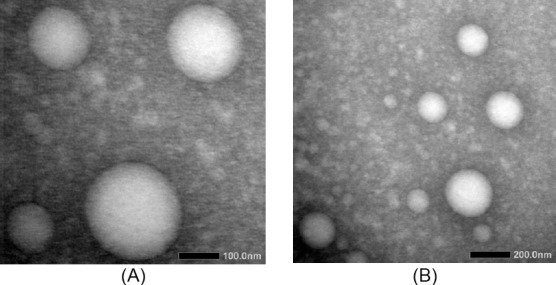
The Transmission Electron Micrograph of nanoemulsion droplets with magnification of 40,000 times (A) and 20,000 times (B).

Figure 2 shows the morphology of curcumin nanoemulsion. The droplets were spherical and fairly uniform in size. This finding is supported by our study on droplet size, where the polydispersity index was low (<0.5).

### Entrapment Efficiency and Loading Capacity Analysis

Entrapment efficiency of curcumin nanoemulsion system was found to be 89.89 ± 8.18%. The solubility of curcumin in the oil phase is an important factor in determining the entrapment efficiency of curcumin. The solubility of curcumin in the GMO 40, Tween 20, and the PEG 400 were reported as 0.154 mg/mL, 60 mg/mL, and 0.512 mg/mL, respectively [11]. Rachmadi (2014) stated that the addition of curcumin at a higher amount decreased the efficiency of entrapment due to the limited capacity of nanoemulsion system to encapsulate the active substance.

In this study, entrapment efficiency of 10 mg of curcumin in 1 g of oil phase was 90.6 ± 9.2 %, indicating minimum loss of unloaded curcumin.

### Zeta potential

Zeta potential indicates the charactheristic of the globule surface. Sufficient value of zeta potential is required (± 30 mV) to ensure a high energy barrier against coalescence of the dispersed globules. However, low zeta potential was obtained in this study, being −0.12 ± 0.50 mV. This is most likely due to the nature of Tween 20 as a nonionic surfactant that covered the surface of droplets. However, the low value of zeta potential is not always an indication of a decrease in the repulsive power or stability [9].

### In Vitro Antihypertensive Activity Assay

**Fig. 3:**
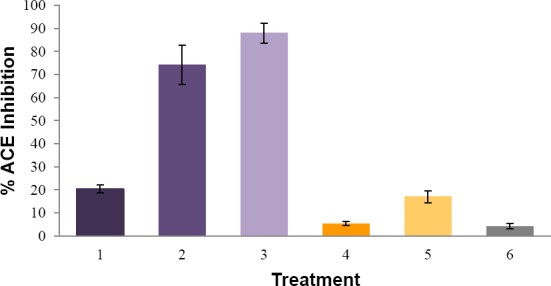
Average inhibition rate of ACE by captopril 3.1 ng/mL (1), captopril 3.6 ng/mL (2), captopril 4.1 ng/mL (3), curcumin 2 mg/mL (4), curcumin nanoemulsion 2 mg/mL (5), unloaded nanoemulsion (6). All measurements were performed in triplicates.

ACE inhibitory activity of curcumin nanoemulsion (2 mg/mL) was determined and compared to captopril in various concentrations: 3.1 ng/mL, 3.6 ng/mL, 4.1 ng/mL, and pure curcumin (2 mg/mL). Their respective rates of ACE inhibition were 17.021 ± 2.611 %, 20.339 ± 1.792 %, 74.157 ± 8.464 %, 87.850 ± 4.368 %, and 5.344 ± 0.820 %, as shown in Figure 3. Unloaded nanoemulsion exhibited ACE inhibition as much as 4.255 ± 1.202 %. This value was used as a correction factor.

The rate of ACE inhibition demonstrated by curcumin was significantly lower than the positive control, captopril, in all concentrations. This means that curcumin had no potential effect as an ACE inhibitor. The fact that curcumin has been reported to have an effect of lowering the blood pressure suggests that the mechanism of action of curcumin in decreasing blood pressure is different from ACE inhibitors. Shimatsu, et.al. (2012) in his study compared the efficacy of curcumin and enalapril (an ACE inhibitor) for treating heart failure on rats with myocardial infarction. They found that therapeutic effect of curcumin was equal to enalapril in alleviating heart failure. In addition, the activity of curcumin in combination with enalapril was found to be higher.

In this study, curcumin nanoemulsion showed higher ACE inhibition rate in comparison with pure curcumin. This finding shows that nanoemulsion carrier system can enhance the inhibition activity of curcumin by improving its solubility.

### In Vitro Antihypercholesterolemic Activity Assay

**Fig. 4:**
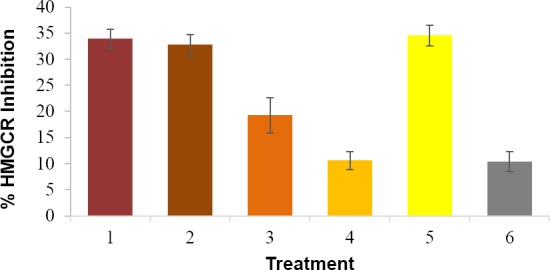
Average HMGCR inhibitory activity of pravastatin 1 mg/mL (1) curcumin 10 mg/mL (2), curcumin 3 mg/mL (3), curcumin 2 mg/mL (4), curcumin nanoemulsion 2 mg/mL (5), and unloaded nanoemulsion (6).

Antihypercholesterolemic activity was interpreted by inhibition rate of HMG-CoA reductase (HMGCR), as shown in Figure 4. The inhibition rate showed by curcumin nanoemulsion (2 mg/mL) was 34.508 ± 1.959 %, while pure curcumin at similar concentration only showed 10.538 ± 1.703 % inhibition. In order to examine the relationship between inhibition activity and curcumin concentration, the assay was performed for pure curcumin in PEG-water 1:1 at concentration of 10 mg/mL and 3 mg/mL. The rate of HMGCR inhibition observed were 32.676 ± 2.011 % and 19.240 ± 3.383 %, respectively. Inhibition activity of pravastatin (1 mg/mL) was 33.897 ± 1.875 %. Unloaded nanoemulsion inhibited HMGR activity by 10.385 ± 1.916 %. Therefore, the corrected inhibition activity of curcumin nanoemulsion was 24.123%. This figure was significantly higher than that of pure curcumin at concentration of 2 mg/mL, 3 mg/mL and 10 mg/mL.

**Fig. 5:**
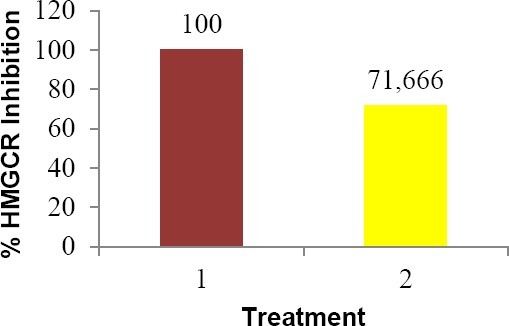
HMGCR Inhibitory activity of curcumin nanoemulsion (2) compared to pravastatin (1). The values are obtained after corrections.

The comparison between curcumin and nanoemulsion in HMGCR inhibition is shown in Figure 5. Pravastatin is stated to inhibit HMGCR at 100%, while in this study the inhibition was 33.897 ± 1.875 %. Corrections have been made to determine the actual inhibition activity of curcumin nanoemulsion. It was found that the inhibitory activity of curcumin nanoemulsion after corrections was 71.166%.

HMG-CoA reductase is an enzyme involved in the biosynthesis of cholesterol in the liver. *In vivo* study showed that curcumin can inhibit the activity of hepatic HMG-CoA and decrease the activity of HMGR gene that codes for HMG-CoA enzyme in the liver [2].

Curcumin is also known to have antihiperlipidemic activity by suppressing the accumulation of triglycerides and cholesterol in the liver. Curcumin can increase the expression of PPARα, the gene that play a role in the regulation of fatty acid oxidation. Curcumin also increases the transcription of LXRα, which regulates CYP7A1 (encoding cholesterol-7a-hydroxlylase). Cholesterol-7a-hydroxlylase plays role in the conversion of cholesterol into bile acids before it is excreted. Curcumin can also suppress the formation of atherosclerotic lesions in mice which is fed with atherogenic food, shown by a decline in the atherogenic indicator and an increase in the percentage of HDL / total cholesterol [2].

## Experimental

Curcumin (PT. Phytochemindo Lestari, Indonesia), glyceryl monooleate 40 (GMO 40, PT. Trinity Arthamakmur, Indonesia), polysorbate 20 (Tween 20, Sigma Aldrich), polyethylene glycol 400 (PEG 400, PT. BRATACO, Indonesia), dimethyl sulfoxide (DMSO, Merck, Germany), deionized water (Laboratory of Chemistry, Bandung Institute of Technology, Indonesia), sodium borate, sodium chloride (NaCl), hydrochloric acid (HCl), ethyl acetate and distilled water (IPHA Laboratories, Indonesia), ACE kit (Sigma Aldrich), HMG-CoA reductase kit (Sigma Aldrich).

### Curcumin Nanoemulsion Preparation using Self-Nanoemulsification (SNE) Method

Preparation of curcumin nanoemulsion was performed using SNE method that has been developed by Rasaputri (2010). Initially, curcumin (10 mg per 1 g oil phase) was dissolved in GMO under a constant stirring for 15 minutes at 100 rpm. Tween 20 was added and the mixture was stirred for another 15 minutes. PEG 400 as cosurfactant was then added, followed by two-hour stirring. The ratio of GMO 40 : Tween 20 : PEG 400 was 1:8:1. The mixture was then ultrasonicated in a sonicator bath for an hour. Aqua deion as the aqueous phase was added with a ratio of oil-to-water phase of 1: 5. Nanoemulsion was formed spontaneously after a mild stirring.

### Nanoemulsion Characterization

#### Droplet Size and Size Distribution Measurement

Droplet size and polydispersity index were measured using Photon Correlation Spectroscopy (Delsa™ Nano C Particle Analyzer, Beckman Coulter).

#### Morphology of nanoemulsion globule

The morphology of nanoemulsion droplet was analyzed using Transmission Electron Microscopy (TEM). Ten microliters of nanoemulsion was placed on a specimen plate and allowed to dry. A 400 mesh grid tool was placed on top of the specimens and the preparation was allowed to stand for a minute. Any residue of nanoemulsion remaining on the grid was cleaned using a filter paper. Ten microliters of uranyl acetate was dropped on top of the grid and the excessive solution was removed by using a filter paper. The grid was left for 30 minutes to dry and the microscopic image was captured.

#### Entrapment Efficiency and Loading Capacity

Nanoemulsion was centrifuged for 20 minutes at 14000 rpm. The precipitate formed represents the free curcumin while encapsulated curcumin remained in the supernatant. The amount of curcumin in supernatant and precipitate were determined by using UV-Visible spectrophotometry at a wavelength of 430 nm. A calibration curve was made using curcumin in DMSO, ranging from 1 to 8 mg/mL. Entrapment efficiency was then determined using the formula in Equation 1, while loading capacity was determined using the formula in Equation 2,


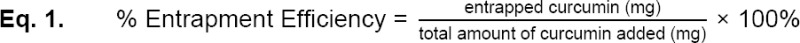



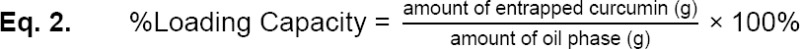


#### Zeta Potential

Zeta potential was determined by using electrophoretic light scattering (Delsa™ C Nano Particle Analyzer, Beckman Coulter).

### In Vitro Antihypertensive Activity Assay

Antihypertensive activity was represented by the ability of curcumin to inhibit ACE. ACE inhibition activity was measured by a UV spectrophotometer based on the rate of formation of hippuric acid from hippuryl-L-histidyl-L-leusine (hhl) catalyzed by ACE [12].

Fifty microliters of ACE (25 mU/mL) was incubated with 50 µL of the test solution at a temperature of 37°C for 10 minutes. Subsequently, 150 µL of substrate solution (8.3 mM of HHL in 50 mM sodium borate buffer containing 0.5 M NaCl, pH 8.3) was added and the mixture was for 30 minutes at 37°C. The reaction was stopped by adding 250 µL of 1.0 M HCl. Then, 0.5 mL of ethyl acetate was added and the mixture was centrifuged at 800 ×g for 15 minutes. A total of 0.2 mL of the upper layer was collected into a test tube and evaporated under vacuum condition. Hippuric acid which was formed was dissolved in 1 mL of distilled water and the absorbance was measured spectrophotometrically at 228 nm. Captopril was used as the standard at a concentration of 3.6 ng/mL. The rate of ACE inhibition was calculated by the formula in Equation 3,


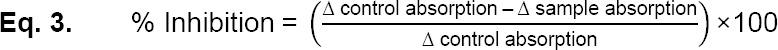


### In Vitro Antihypercholesterolemic Activity Assay

Antihipercholesterolemic activity was determined by measuring the decrease in NADPH absorbance by 3-hydroxy-3-methylglutaryl-CoA reductase (HMGR) in the presence of HMG-CoA substrate. Firstly, a blank solution, non-inhibitory control, inhibition control (1 mg/mL of pravastatin), and test solutions were prepared. Test solutions included pure curcumin in PEG: water 1:1 with a concentration of 10 mg/mL, 3 mg/mL, and 2 mg/mL, curcumin nanoemulsion with a concentration of 2 mg/mL, and unloaded nanoemulsion. A buffer solution was added to all solution (184 µL for the blank solution, 181 µL for the inhibition control and the test solution, 182 µL for non-inhibitory control). One milliliter of pravastatin was added into inhibition control. The reaction mixt containing 4 mL of NADPH, 12 mL of HMG-CoA, and 2 mL of HMGR was added to the test solution, non-inhibitory control, and inhibition control. All of the mixtures were shaken for at least 10 seconds before measuring the absorbance. Measurement was performed using UV spectrophotometer at 340 nm. The temperature of all preparations was maintained at 37°C. Readings were measured every 20 seconds for 10 minutes. Inhibition activity was calculated using the formula in Equation 4,


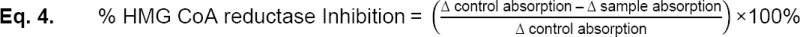


### Data Evaluation

All data shown were presented in the form of mean ± standard deviation. Data were obtained from three replications (n = 3). Statistical analysis was performed using t-test unpaired student and one way ANOVA.
